# PReS-FINAL-2230: A prospective evaluation of a cohort of patients with PFAPA syndrome

**DOI:** 10.1186/1546-0096-11-S2-P220

**Published:** 2013-12-05

**Authors:** M Cattalini, I Bosio, A Meini, P Cancarini, M Berlucchi, G Savoldi

**Affiliations:** 1 Pediatric Immunology and Rheumatology, Pediatric Clinic, University of Brescia, Brescia, Italy; 2 Pediatric Otorynolaryngology, University of Brescia, Brescia, Italy; 3 Pediatric Genetics Laboratory, Pathology Department, University of Brescia, Brescia, Italy

## Introduction

PFAPA (Periodic Fever with Aphtous stomatitis, Pharyngitis and cervical Adenitis) is a periodic syndrome described for the first time in 1987 by Marshal et al. In 1999 the diagnostic criteria were formulated by Thomas.

## Objectives

prospective evaluation of a cohort of PFAPA patients, to better describe clinical characteristics and therapeutic response.

## Methods

all patients receiving a PFAPA diagnosis between 1999 and 2012 were prospectively evaluated. Sex, age at onset, age at diagnosis, family history, clinical characteristic of the febrile episodes and associated symptoms, prodromes, therapy, therapy response and age at resolution were collected.

## Results

In our cohort (148 males and 120 females) fever began at 26.2 ± 24 months of age. 8% of the patients had an onset after the fifth year of life, but all other Thomas criteria were met. A family history was present in 39.6% of patients. Mean duration of PFAPA episodes was 4 ± 1.6 days, and a mean interval between episodes 27.9 ± 11 days. Most common symptoms with fever were pharyngitis (95.5%), cervical adenitis (63.8%), stomatous aphtosis (38.4%), abdominal pain (32%). Prodromes, such as irritability, nausea and headache were present in 10% of patients. All patients received treatment with oral steroids, using a single administration of 1 mg/kg of prednisone or prednisone equivalent, the first day of fever. In all patients steroids were effective and only 13% of them experienced a free-interval shortening, without the perceived need to stop the steroids for this reason. There was no difference in the studied parameters between the population who experienced a free-interval shortening and the population in which this event was not registered.

In 144 children resolution occurred, in 58% of children spontaneously and in 42% after tonsillectomy. Mean disease duration was 40 ± 63 months, medium age at resolution 67.7 ± 66 months. Tonsillectomy was efficacious in 60/62 patients. The tonsillectomy was done after a mean period of 36 months from disease onset.

At multivariate regression analysis disease resolution was independently associated to age onset (β = 1.011 95%CI 1.000-1.022, p = 0.05) and to tonsillectomy (β = 0.022 95%CI 0.005-0.092 p = 0.001).

## Conclusion

PFAPA is the most common cause of periodic fever in children, however our study confirms that the 5 year of age at disease onset criterion is too strict. Symptoms other than the ones from the classic description, such as abdominal pain, could have clinical relevance. Prodromes are quite common and useful in differentiate the typical PFAPA attack from other episodes of fever. Oral steroids are, in our opinion, the therapy of choice and the free-interval shortening is not perceived as a clinically relevant issue. It is not possible to predict which patients would present this effect. Tonsillectomy is very effective, but should be reserved to a very selected group of patients and with ad adequate period of follow-up before the surgery. Age at onset seems to inversely correlate with disease duration.

## Disclosure of interest

None declared.

